# Understanding mechanisms of *Polygonatum sibiricum*-derived exosome-like nanoparticles against breast cancer through an integrated metabolomics and network pharmacology analysis

**DOI:** 10.3389/fchem.2025.1559758

**Published:** 2025-06-06

**Authors:** Tingwen Ming, Yang Yang, Bing Shang, Zhihao Li, Fangling Ren, Lun Wu, Shuya Zhang, Jun Zhu, Qinhua Chen, Jingjian Liu

**Affiliations:** ^1^ Department of Pharmacy, Sinopharm Dongfeng General Hospital, Hubei University of Medicine, Shiyan, China; ^2^ Department of Pharmacy, Shenzhen Bao’an Authentic TCM Therapy Hospital, Shenzhen, China; ^3^ Department of Pharmacy, National Cancer Center/National Clinical Research Center for Cancer/Cancer Hospital, Chinese Academy of Medical Sciences and Peking Union Medical College, Beijing, China

**Keywords:** *Polygonatum sibiricum*, exosome-like nanoparticles, breast cancer, metabolomics, network pharmacology, bioinformatics

## Abstract

**Background:**

*Polygonatum sibiricum* has a long history of medicinal and edible usages, and has attracted widespread attention from researchers due to its rich pharmacological activities. Research has found that plant-derived exosome-like nanoparticles (PELNs) have enormous potential in the field of biomedicine, such as serving as natural nanomedicines to treat diseases or as carriers for drug delivery. However, there are no studies on *P. sibiricum*-derived exosome-like nanoparticles (PSELNs) against cancer.

**Methods:**

This work used ultracentrifugation to extract the PSELNs and characterized them using transmission electron microscopy (TEM), nanoparticle tracking analysis (NTA), and dynamic light scattering (DLS). Proteomics and metabolomics were used to analyze the components of the PSELNs, and the Herb database was used to screen for active metabolites. The OMIM and TTD databases were used to analyze active metabolites, and we further speculated that they may have anti-breast cancer (BC) activity. Network pharmacology was used to analyze the possible mechanisms of the PSELNs against BC, mainly including protein-protein interaction (PPI) network analysis for potential targets, gene ontology (GO) for analyzing biological processes, and Kyoto Encyclopedia of Genes and Genomes (KEGG) for analyzing related signaling pathways. After that, the related data of BC was retrieved from the GEO database, and the clinical expression and survival prognosis of the key genes screened by network pharmacology were analyzed by bioinformatics. Molecular docking and Molecular dynamics (MD) simulation were used to verify the binding of active metabolites in the PSELNs with their targets. Finally, the CCK-8 method was used to validate the inhibitory effect of the PSELNs on BC.

**Results:**

Firstly, TEM, NTA, and DLS confirmed that the PSELNs were successfully isolated. Then, Proteomics identified 18 protein components from the PSELNs, including ATP synthase subunit alpha, protein Ycf2, and Mannose/silica acid binding lectin. Metabolomics identified 357 metabolic components from the PSELNs and further screened 23 active metabolites by oral bioavailability (OB), including Sedanolide, Baicalein, and 6-Gingerol, etc. By analyzing 23 active metabolites, it was speculated that the PSELNs may have pharmacological activity against BC. After that, network pharmacology was used to screen 23 key targets of the PSELNs against BC. KEGG and GO enrichment analysis showed that the MAPK signaling pathway, PI3k-Akt signaling pathway, and AMPK signaling pathway were involved in the anti-BC effect of the PSELNs. Through bioinformatics analysis of 23 key targets in BC clinical samples, it was found that, except for ESR1, which was significantly upregulated, CAV1, FGF2, and PPARG were all significantly downregulated. The expression levels of 4 targets were positively correlated with survival status. These 4 targets were validated by molecular docking and MD simulation with active metabolites in the PSELNs, and it was found that the binding of sedanolide and ESR1, Baicalein and PPARG, and 6-Gingerol and ESR1 can remain stable. Finally, The inhibitory effect of the PSELNs on BC cells (MDA-MB-231) was validated by the CCK-8 method.

**Conclusion:**

This was the first time that the PSELNs have been studied against cancer. It was verified that they have anti-BC activity, and the mechanism may be related to targets such as ESR1 and PPARG, regulated by active metabolites in the PSELNs. This work laid a foundation and reference for more follow-up related research studies.

## 1 Introduction

In 2020, female breast cancer (BC) was recorded to be the main cause of global cancer incidence rate, and also ranks first in female cancer incidence rate and mortality (Sung et al., 2021). Although with the development and application of imaging technology such as breast ultrasound and MRI, the current management effect of BC is still very unsatisfactory (Rossi et al., 2019). Many factors, such as tumor type, histological grade, lymph node metastasis, estrogen receptor (ER), and progesterone receptor (PR), affect the treatment response and prognosis of BC (Jokar et al., 2021). Surgical treatment, radiotherapy, and chemotherapy are still the basic methods to treat BC, but with advanced BC of up to stage IV, its 5-year survival rate is only 28% (SEER 18, 2013–2019) (National Cancer Institute, 2021).

Traditional Chinese medicine (TCM) has the unique advantages of multi-target and minimal side effects, and is often used as a supplement and alternative therapy in cancer treatment (Yang et al., 2021). *Polygonatum sibiricum* belongs to the order Liliaceae, Liliaceae and genus *P. sibiricum*. It is mostly distributed in temperate regions of the northern hemisphere, including China, Russia, Europe, North America, and other places (Zhao et al., 2018). As a typical plant with medicinal and edible properties, it has a long history of application. Modern research has found that *P. sibiricum* has pharmacological effects such as antioxidant, anti-aging, immune regulation, anti-tumor, blood glucose lowering, and blood lipid regulation (Liu R. et al., 2024). It has been widely developed and utilized in the fields of food, medicine, and health products (Xiaowei et al., 2019).

Exosomes are nanovesicles with a diameter of 40–150 nm secreted by cells to the outside of the cell and play an important role in cell communication (Boccia et al., 2022). Plant-derived exosome-like nanoparticles (PELNs) are gaining significant interest in drug development owing to their natural richness in bioactive components, including proteins, lipids, RNA, and small molecules (Kim et al., 2022). For example, ginseng-derived exosome-like nanoparticles can exert anti-glioma effects by active blood-brain-barrier penetration and tumor microenvironment modulation (Kim et al., 2023). Yam-derived exosome-like nanovesicles can stimulate osteoblast formation and prevent osteoporosis in mice (Hwang et al., 2023). Compared to exosomes derived from mammals, PELNs have the advantages of wide availability, low cost, and rich pharmacological activity (Mu et al., 2023). In addition, PELNs have great potential as nanocarriers for drugs (Dad et al., 2021). At present, research on the pharmacological activity of *P. sibiricum* mainly focuses on polysaccharides, saponins, and extracts (Zhao et al., 2022). However, there is almost no research on the *P. sibiricum*-derived exosome-like nanoparticles (PSELNs), and their composition and pharmacological activity still need to be explored (Luo et al., 2022).

Proteomics and metabolomics are widely used in the field of medicine. Metabolomics is an efficient technique for studying various small-molecule compounds within substances, with wide applications in plant component analysis (Hazrati et al., 2022). The commonly used identification method at present is the combination of chromatography and mass spectrometry to achieve the separation and identification of substances, among which ultra-high liquid chromatography mass spectrometry can achieve accurate qualitative and quantitative analysis of substances (Wang K. et al., 2023). Network pharmacology serves as an effective approach to elucidate drug mechanisms, addressing the complexity of TCM components and their pharmacological features, which involve multiple targets and diverse signaling pathways (Zhao et al., 2023). Network pharmacology combines systems biology and systems pharmacology to achieve multi-level and systematic explanations of drug mechanisms of action (Zhang et al., 2023). Molecular docking technology and molecular dynamic (MD) simulation are often used in conjunction with network pharmacology to simulate the binding of molecular drugs to targets (Sun et al., 2023).

**FIGURE 1:**
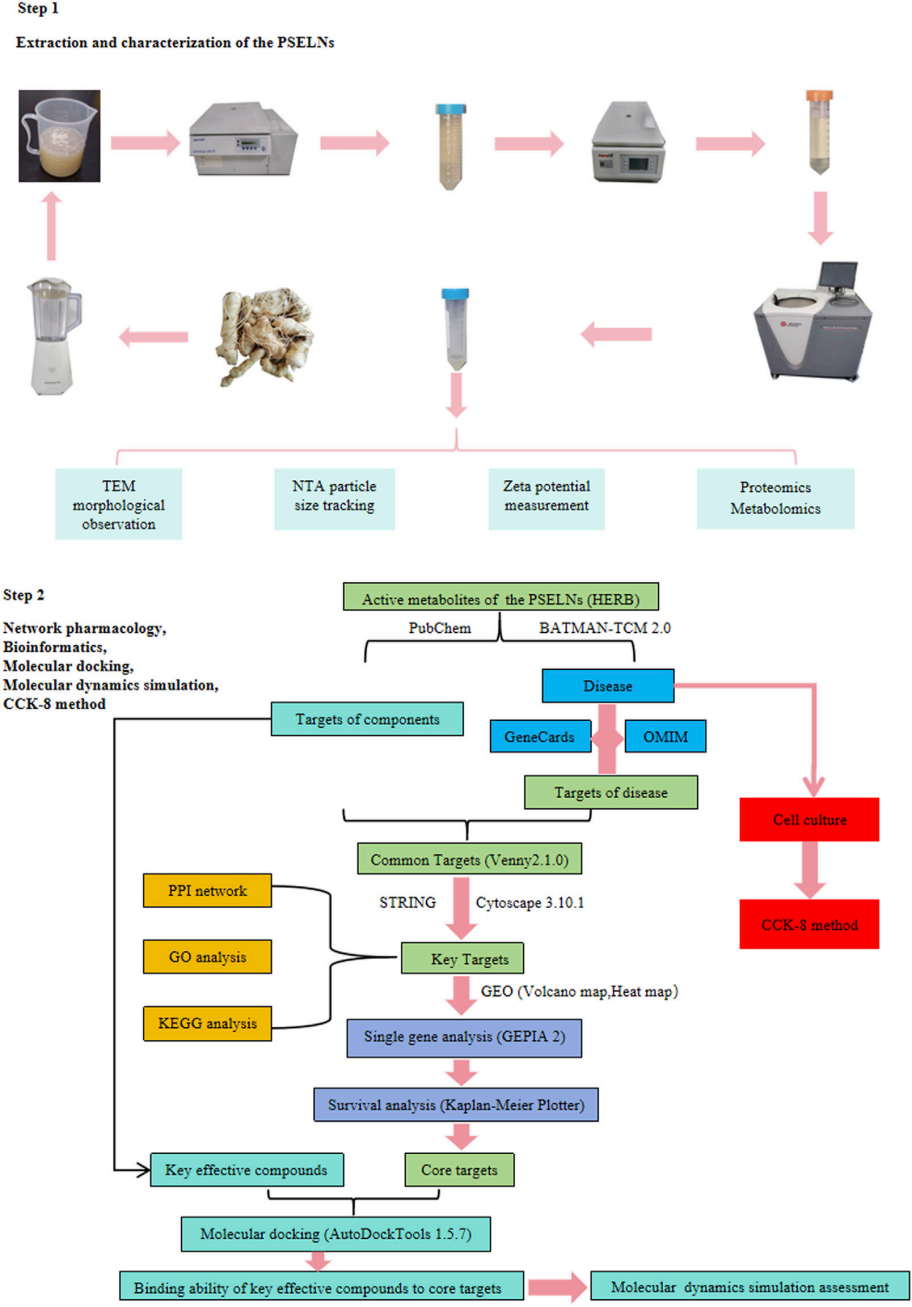
General flow chart.

In this work, we used ultracentrifugation to successfully extract exosome-like nanoparticles from the roots of *P. sibiricum* and analyzed their composition through proteomics and metabolomics. Then, the pharmacological effects of its active metabolites were explored through network pharmacology methods to achieve a preliminary evaluation of the pharmacological effects of the PSELNs. Combined with molecular docking and MD simulation, the functional components and main targets were validated, providing ideas and evidence for subsequent research on the PSELNs. Finally, The inhibitory effect of the PSELNs on BC cells (MDA-MB-231) was validated by the CCK-8 method. The detailed workflow is shown in Figure 1.

## 2 Materials and methods

### 2.1 Isolation and characterization of the PSELNs

#### 2.1.1 Isolation of the PSELNs

*Polygonatum sibiricum* was excavated and refrigerated for transportation, followed by immediate extraction of the PSELNs. Firstly, the cleaned root of *P. sibiricum* was cut into small pieces and placed in a juicer. After adding an appropriate amount of phosphate-buffered saline (PBS, pH 7.4), it was ground for 5 min. Then, the filtrate was filtered through gauze (24–36 mesh, Winner Medical Co., Ltd.) and centrifuged at 4,000 rpm for 90 min to remove large granular cell debris and residual cellular components. Subsequent centrifugation at 10,000 rpm for 90 min was used to remove medium particles (e.g. microvesicles) and smaller cell debris. Thereafter, centrifugation at 12,000 rpm for 90 min was performed to remove smaller protein aggregates. Subsequently, the supernatant was filtered through a 450 nm filter membrane and centrifuged at 100,000 g for 2 h at 4°C (Optima XE100 ultracentrifuge, Beckman Coulter) to enrich the PSELNs. The supernatant was discarded, and the pellet was dissolved in PBS. Finally, after filtration through a 220 nm membrane, the PSELNs were obtained and stored in a −80°C refrigerator for future use.

#### 2.1.2 Characterization of the PSELNs

Transmission electron microscopy (TEM) was used for the morphological characterization of the PSELNs. The PSELNs were dropped onto a copper mesh and left to air dry. After staining with a 3% phosphotungstic acid solution, they were left to air dry again and photographed using a TEM (HT7800).

Nanoparticle Tracking Analysis (NTA) was used to evaluate the particle size distribution of the PSELNs. The PSELNs were diluted 50 times and tested using Nanosight NS300 (Malvern).

The Zeta potential of the PSELNs was used for characterization. Particle size and potential analyzer (Malvern, Nano ZS90) was used to measure the PSELNs potential.

### 2.2 Proteomics

Acetonitrile was purchased from Thermo Fisher Scientific (Waltham, MA, United States). All solvents were LC-MS grade. Dimethyl sulfoxide, sodium deoxycholate, and 2-chloroacetamide were purchased from Sigma-Aldrich (St. Louis, MO, United States). Trypsin was purchased from SignalChem.

#### 2.2.1 Test sample preparation

After detecting the protein concentration of the PSELNs using the BCA method, the protein content in each sample was fixed, and the solution (1% SDC/100 mM Tris HCl (pH 8.5)) was used to bring the volume to the same volume. Protein reduction and alkylation were performed with TCEP and CAA at 60°C for 30 min. The same volume of ddH_2_O was added to dilute the SDC to less than 0.5%. Trypsin was added at a ratio of 1:50 (enzyme: protein, w/w) for digestion overnight at 37°C. The next day, after the pH value of the solution was lowered to 6.0 by TFA, 12,000 g was centrifuged for 15 min, and a self-made SDB-RPS desalination column was used for peptide purification of the supernatant. The peptide eluate was dried *in vacuo* and stored at −20°C for later use.

#### 2.2.2 Mass spectrometry

All the samples of the PSELNs were analyzed on an Ultimate 3,000 RSLCnano system. Peptide samples were injected into a C18 Trap column (75 μm × 2 cm, 3 μm particle size, 100 Å pore size, Thermo) and separated in a reversed-phase C18 analytical column packed in-house with ReproSil-Pur C18-AQ resin (75 μm × 25 cm, 1.9 μm particle size, 100 Å pore size). Mobile phase A (0.1% formic acid, 3% DMSO, 97% H_2_O) and mobile phase B (0.1% formic acid, 3% DMSO, 97% ACN) were used to establish the separation gradient at a flow rate of 300 nL/min. The MS was operated in data-dependent acquisition (DDA) top 20 mode with a full scan range of 350–1,500 m/z.

#### 2.2.3 Database retrieval

The MS raw data of the PSELNs was analyzed with MaxQuant (1.6.6.0) using the Andromeda database search algorithm.

### 2.3 Metabolomics

Methanol, acetonitrile, formic acid, and isopropyl alcohol were purchased from ANPEL. All solvents were LC-MS grade. And ultra-pure water in-house prepared using a Milli-Q water purification system (Millipore, Bedford, MA, United States).

#### 2.3.1 Sample processing

First, 0.5 mL of the PSELNs was slowly thawed at 4°C and placed in centrifuge tubes. Then, a double volume of the extraction solution (methanol/acetonitrile, 1:1, v/v) was added and vortexed on a vortex oscillator (Thermo Fisher) mixer at 3,000 rpm for 60 s to mix well. Subsequently, after 30 min of low-temperature ultrasonic extraction, the mixture was centrifuged at 12,000 rpm for 10 min at 4°C. After standing at −20°C for 1 h, the mixture was centrifuged at 12,000 rpm for 10 min at 4°C, and the supernatant was dried in a vacuum. Finally, 0.1 mL of 50% acetonitrile solution was added and homogenized. The mixture was centrifuged at 12,000 rpm for 10 min at 4°C, and the supernatant was collected for detection.

#### 2.3.2 UPLC conditions

The sample extracts were analyzed using a UPLC-Orbitrap-MS system, which consisted of a Vanquish UPLC and an HFX MS. The UPLC conditions were as follows: a Waters HSS T3 column (100 × 2.1 mm, 1.8 μm) was used at a temperature of 40°C; the flow rate was set to 0.3 mL/min; the injection volume was 2 μL; and the solvent system comprised of Milli-Q water with 0.1% formic acid as phase A and acetonitrile with 0.1% formic acid as phase B. The gradient program was as follows: 0 min (100% phase A, 0% phase B), 1 min (100% phase A, 0% phase B), 12 min (5% phase A, 95% phase B), 13 min (5% phase A, 95% phase B), 13.1 min (100% phase A, 0% phase B), and 17 min (100% phase A, 0% phase B).

#### 2.3.3 LC-MS/MS analysis

The Q Exactive HFX Hybrid Quadrupole Orbitrap mass spectrometer, equipped with a heated ESI source from Thermo Fisher Scientific, was used to record HRMS data using the Full-ms-ddMS2 acquisition methods. The ESI source was configured with the following parameters: a sheath gas pressure of 40 arb, an aux gas pressure of 10 arb, a spray voltage of either +3,000 V/−2,800 V, a temperature of 350°C, and an ion transport tube temperature of 320°C. The primary mass spectrometry scanning range was set to 70–1,050 Da, with a resolution of 70,000, while the secondary resolution was 17,500.

### 2.4 Network pharmacology analysis

#### 2.4.1 Screening of active metabolites from the PSELNs

The metabolites of the PSELNs were compared with the active ingredients of the TCM in the Herb database (http://herb.ac.cn/), and screened under the condition of oral bioavailability (OB) ≥ 30% (Xiao et al., 2022). The screened active ingredients were the active metabolites of the PSELNs.

#### 2.4.2 The PSELNs active metabolite targets and disease prediction

The PubChem CID and related data of the active metabolite were found in the PubChem database (https://pubchem.ncbi.nlm.nih.gov/). Then, in the MATMAN-TCM database (HomePage-BATMAN (ncpsb.org.cn)), the enriched target and diseases of the active metabolite were queried by the PubChem CID number, and the screening criteria was Score ≥20 and Adjusted *P*-value <0.5.

#### 2.4.3 The PSELNs-target-disease analysis

BC was selected as the research object from the disease enrichment results. Firstly, through the GeneCards database (https://www.genecards.org/) and the OMIM database (https://www.omim.org/), target genes of BC were downloaded. The target genes of BC obtained from the two databases were merged, which were the potential targets of BC. Afterwards, the intersection of the enriched targets of active metabolites and the enriched targets of diseases were taken as the common target of both by Venny 2.1.0 (http://www.liuxiaoyuyuan.cn/). Then, in the STRING database (https://cn.string-db.org/), the potential targets were analyzed with “*Homo sapiens*” as the object, and the gene interaction network data was downloaded. The data was visualized in Cytoscape software (3.10.1), and the key targets were topologically analyzed through the Centiscape plug-in. Using “Betweenness, Closeness, Degree” as the screening criterion, the targets larger than the central value were analyzed for intersection. The final intersection target was the key targets of the selected the PSELNs against BC.

#### 2.4.4 GO and KEGG enrichment analysis

The key targets were placed in the DAVID database (https://david.ncifcrf.gov/) for enrichment analysis using “*H. sapiens*” as the object. In the Gene Ontology (GO) section, biological process (BP), cellular component (CC), and molecular function (MF) were selected for analysis. The data was downloaded and sorted in descending order based on the Gene ratio. Under the condition of *P* < 0.05, the top 10 GO terms were selected as the research objects and visualized in the bioinformatics database. Similarly, the Kyoto Encyclopaedia of Genes and Genomes (KEGG) pathway analysis were conducted in the Pathways section, with the top 20 pathways as the research subjects.

#### 2.4.5 Construction of the active metabolite-target-pathway-disease network diagram

The key targets were analyzed in the STRING database using “*H. sapiens*” as the object, and the Protein-Protein Interaction (PPI) network diagram was downloaded. Then, the data of active metabolites, key targets, pathways, and BC were combined and processed, and the active metabolite-target-pathway-disease network diagram was drawn in Centiscape (2.2).

### 2.5 Bioinformatics analysis

Gene chip GSE233242 (n = 42) was selected as the research object in the GEO database, which contains both BC tissue samples (Tumor group) and adjacent tissue samples (Normal group). GEO2R was used for processing gene chip data, and data analysis was performed through the limma package in R. After obtaining gene expression data, the expression of key genes screened by network pharmacology in the gene chip was plotted as volcano maps and cluster heat maps. *P* < 0.05 and |logFC| ≥1.5 (Vyas et al., 2025) were used as criteria for screening targets. The GEPIA2 database (http://gepia2.cancer-pku.cn/) was used to analyze the single gene expression of the screened gene, the Kaplan-Meier Plotter online database (https://kmplot.com/analysis/) was used to analyze its prognosis in BC, and a survival analysis curve was drawn.

### 2.6 Molecular docking

The 3D structures of small-molecule compounds were retrieved from the PubChem database, while the 3D structures of proteins were sourced from the PDB database (https://www.rcsb.org/). AutoDockTools (1.5.7) was used for molecular docking simulations, and the Pymol software was used for result visualization.

### 2.7 Molecular dynamic (MD) simulation

MD simulation was used to evaluate the reliability of molecular docking results. The simulation was carried out through the Gromacs2022 program. The FAFF force field was used for small molecules, and the AMBER14SB force field and TIP3P water model were used for proteins. After the composite system of proteins and small molecules were constructed, the MD simulation was performed under constant temperature, pressure, and periodic boundary conditions. The simulation temperature was controlled to 298 K, and the pressure was 1 bar. NVT versus NPT equilibrium simulations at 100 ps were performed, and MD simulations at 100 ns were performed on the complex system, with conformation preservation every 10 ps. The simulated trajectories were analyzed using VMD and Pymol.

### 2.8 Cell experiments

#### 2.8.1 Cell culture

MDA-MB-231 cells were purchased from Pricella Biotechnology Co., Ltd. (Wuhan, China). At 37°C and 5% CO_2_, MDA-MB-231 cells were cultivated in DMEM (CellMax, China) media supplemented with 10% fetal bovine serum (CellMax, China), 1% penicillin and streptomycin (Hyclone, United States).

#### 2.8.2 CCK-8 method

MDA-MB-231 cells (5 × 10^3^ cells/well) were placed in a 96-well plate and incubated in a 37 °C. After 24 h, the PSELNs with different protein concentrations were used to intervene in MDA-MB-231 cells, and the protein concentration of the PSELNs was measured by the BCA method. After continuing to culture for 24 h, the cell survival rate was calculated according to the CCK-8 method instructions.

## 3 Results

### 3.1 Characterization of the PSELNs

**FIGURE 2:**
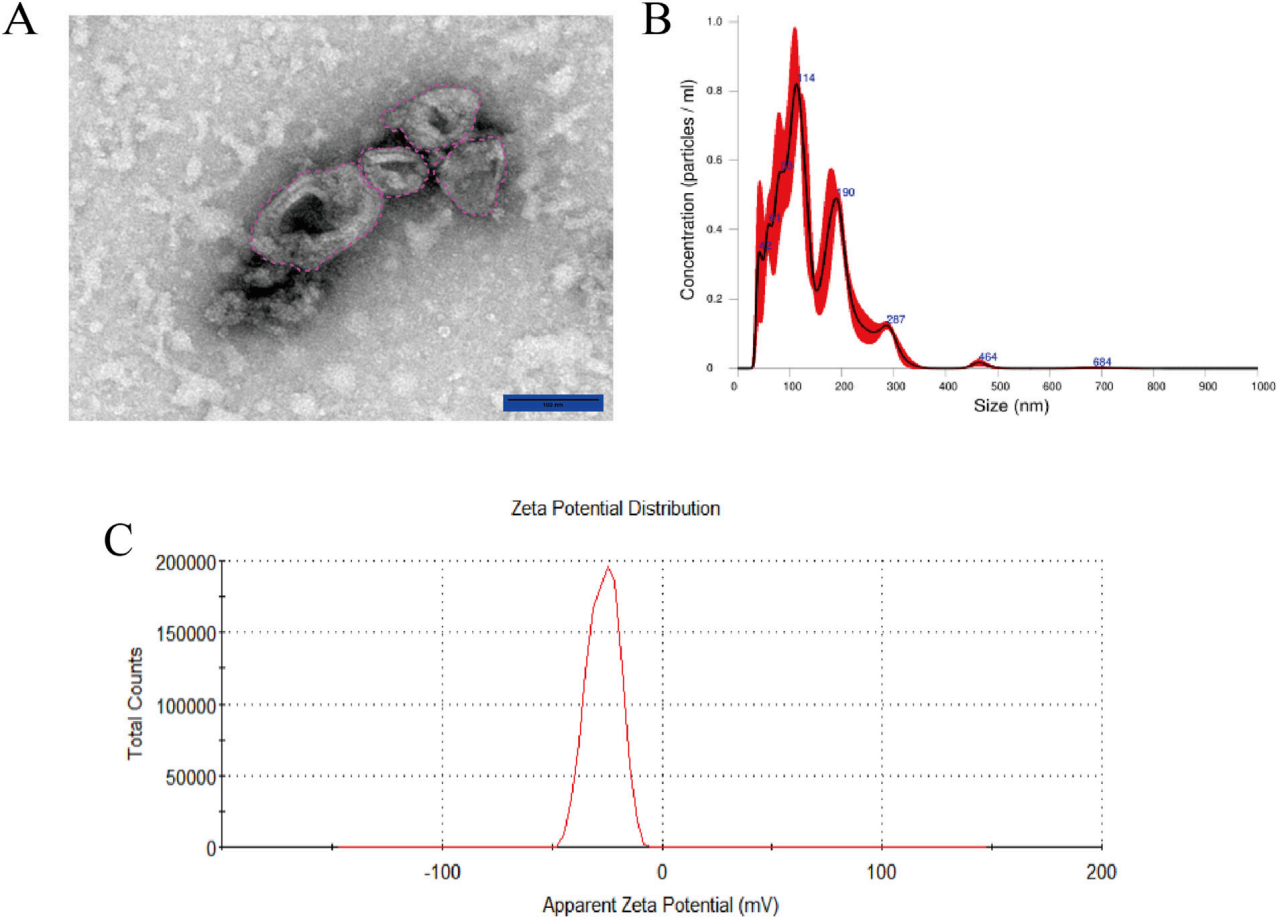
**(A)** The TEM imaging (scale bar: 100 nm); **(B)** The particle size distribution detected by NTA; **(C)** The Zeta potential.

After the PSELNs were extracted from the root of *P. sibiricum* by ultracentrifugation, TEM was used for morphological characterization of the PSELNs. As shown in Figure 2A, the PSELNs have a circular or tea tray-shaped bilayer structure, with a brighter outer layer and a darker inner layer, which is consistent with the morphological characteristics of PLENs reported previously (Luo et al., 2025). Furthermore, NTA was used to measure the particle size distribution of the PSELNs, and the result was shown in Figure 2B. It was found that the average particle size of the PSELNs was 142.4 nm, with the main peak corresponding to a particle size of 114 nm. The concentration of the diluted PSELNs measured was 9.55 ± 0.981 × 10^8^ particles mL^−1^. Most of the detected PSELNs were distributed between 30–150 nm. In addition, the average Zeta potential of the PSELNs was −26.6 mV (Figure 2C). The above results indicate that the PSELNs have been successfully isolated.

### 3.2 Proteomics analysis of the PSELNs

Based on proteomics, 18 proteins were identified from the PSELNs (Table 1), including ATP synthase subunit alpha, protein Ycf2, and Mannose/silica acid binding lectin. The successful detection of these protein components indicates that the PSELNs may play an important role in the growth regulation of *Polygonatum sibiricum*, and are also an important material basis for the pharmacological activity of the PSELNs.

**TABLE 1:** Protein composition of the PSELNs.

Number	Protein IDs	Protein names	Gene
1	H6X2Q5	ATP synthase subunit alpha	atp1
2	A0A0F7C9C0	Ribulose bisphosphate carboxylase large chain	rbcL
3	R4QNF5	Ribulose bisphosphate carboxylase large chain	rbcL
4	A0A7M3V8D5	DNA-directed RNA polymerase subunit beta'	rpoC1
5	A0A0S1RPS6	Protein Ycf2	ycf2
6	A0A290D937	Protein TIC 214	ycf1
7	Q9M654	Ribosome-inactivating protein PMRIPm	RIPm
8	Q8L568	Mannose/sialic acid-binding lectin	-
9	A0AA51RGT7	ATP synthase subunit alpha, chloroplastic	atpA
10	A0A1C9HCC9	ATP synthase subunit beta	atpB
11	A0AA51RJW9	Small ribosomal subunit protein uS11c	rps11
12	I1TJ67	Cytochrome b	cob
13	A0AA51NPN4	DNA-directed RNA polymerase subunit beta''	rpoC2
14	A0AA51RHI1	Small ribosomal subunit protein uS7c	rps7
15	A0AA51RL96	NAD(P)H-quinone oxidoreductase subunit H, chloroplastic	ndhH
16	A0A6B7HE64	Maturase K	matK
17	A0A0S1RQ13	NAD(P)H-quinone oxidoreductase subunit 5, chloroplastic	ndhF
18	A0AA51NP50	Large ribosomal subunit protein uL22c	rpl22

### 3.3 Metabolite analysis of the PSELNs

**FIGURE 3:**
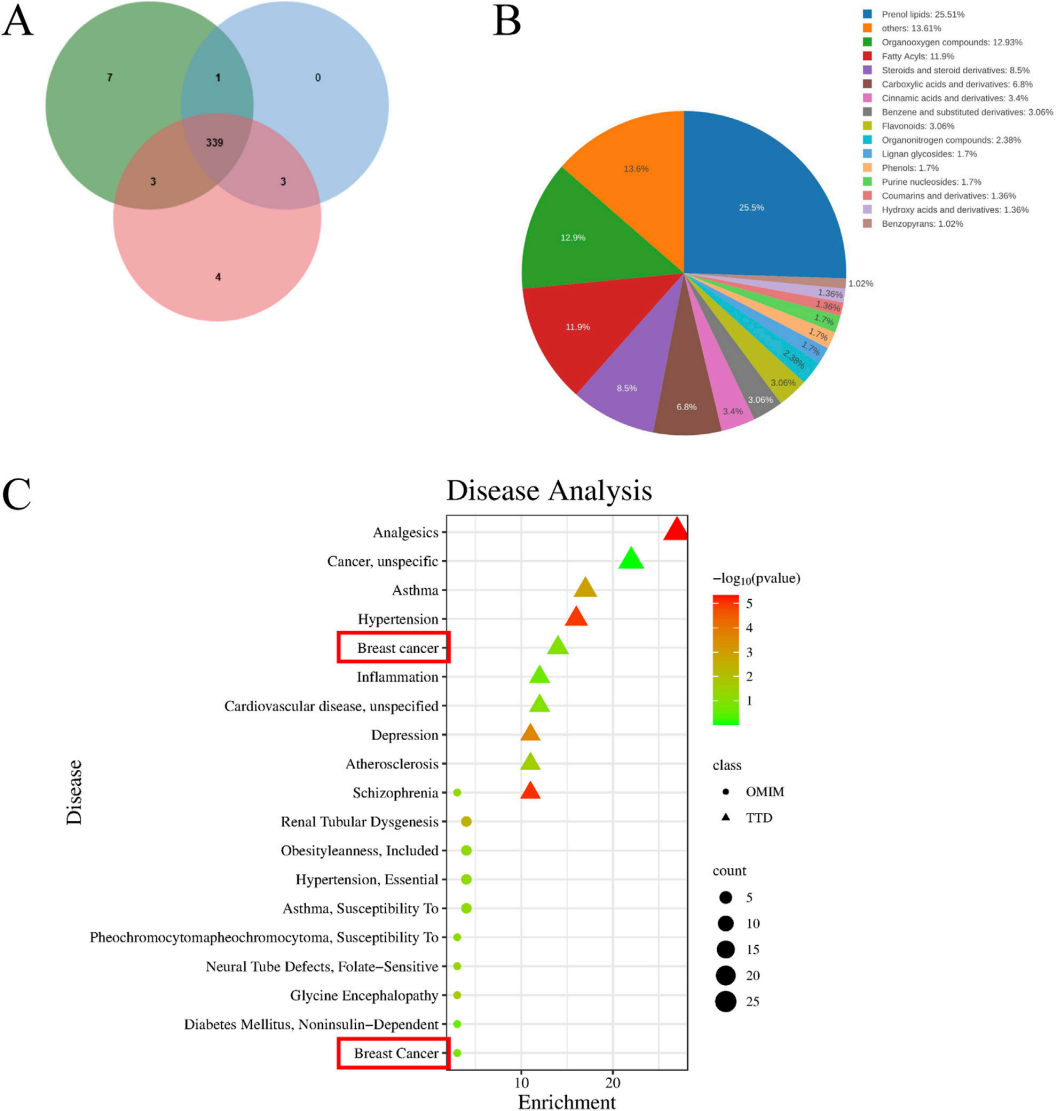
**(A)** The number of metabolites detected in the PSELNs (n = 3); **(B)** Classification of metabolites detected in the PSELNs; **(C)** Enriched disease conditions.

Metabolomics technology based on UPLC-MS/MS was used for metabolite analysis of the PSELNs, and a total of 357 metabolites were identified (Figure 3A), including 139 lipids and their analogs. As shown in Figure 3B, prenol lipids account for 25.51%, steroids and steroid derivatives account for 8.5%, cinnamic acids and derivatives account for 3.4%, flavonoids account for 3.06%, lignan glycosides account for 1.7%, coumarins and derivatives account for 1.36%. To further clarify the active metabolites in the PSELNs, the detected metabolites were compared with the active ingredients in the Herb database and screened under the condition of OB score ≥30%. A total of 23 active metabolites were screened, and they were listed in Table 2.

**TABLE 2:** Selected active metabolites of the PSELNs.

Number	Ingredient name	Ingredient formula	Molecular weight (g/mol)	OB score (%)	PubChem CID
1	Leucinol	C_6_H_15_NO	117.19	72.67,415,654	111,307
2	Syringaldehyde	C_9_H_10_O_4_	182.17	67.0609,961	8,655
3	Adenine	C_5_H_5_N_5_	135.13	62.80,583,588	190
4	Sedanolide	C_12_H_18_O_2_	194.27	62.45,827,708	5,018,391
5	Panaxydol	C_17_H_24_O_2_	260.399	61.66,659,941	126,312
6	Danshenol B	C_22_H_26_O_4_	354.4	57.9,508,753	3,083,515
7	14-deoxyandrographolide	C_20_H_30_O_4_	334.4	56.3,040,973	11,624,161
8	Deoxyandrographolide	C_20_H_30_O_4_	334.4	56.3,040,973	90,476,183
9	Quinic acid	C_7_H_12_O_6_	192.17	55.9,242,283	6,508
10	Cedryl acetate	C_17_H_28_O_2_	264.4	53.93,615,708	13,918,856
11	Vanillin	C_8_H_8_O_3_	152.15	51.99,600,777	1,183
12	Stearidonic acid	C_18_H_28_O_2_	276.4	45.7,751,736	5,312,508
13	Linolenic acid	C_18_H_30_O_2_	278.4	45.00906,591	5,280,934
14	Griffonilide	C_8_H_8_O_4_	168.15	43.15,190,768	100,341
15	Maesanin	C_22_H_34_O_4_	362.5	42.77,499,821	5,384,838
16	Betaine	C_5_H_11_NO_2_	117.15	40.92,229,672	247
17	Eucommiol	C_9_H_16_O_4_	188.22	40.16,705,584	154,373
18	Isovanillic acid	C_8_H_8_O_4_	168.15	39.42,393,771	12,575
19	6-gingerol	C_17_H_26_O_4_	294.4	35.63,854,787	442,793
20	Vanillic acid	C_8_H_8_O_4_	168.15	35.47,235,319	8,468
21	Indican	C_14_H_17_NO_6_	295.29	34.90,438,578	441,564
22	Baicalein	C_15_H_10_O_5_	270.24	33.51,891,869	5,281,605
23	Ethyl brevifolincarboxylate	C_15_H_12_O_8_	320.25	30.8,553,161	5,487,248

The 23 active metabolites selected through screening were enriched for diseases in the MATMAN-TCM database, concerning the OMIM and TTD databases. The top 10 diseases in the enrichment results were shown in Figure 3C. The related diseases enriched in the TTD database include Analgesics, Cancer (unspecific), Asthma, Hypertension, Breast cancer, Inflammation, Cardiovascular disease (unspecified), Schizophrenia, Depression, Atherosclerosis. The relevant diseases enriched in the OMIM database include Renal Tubular Dysgenesis, Hypertension (Essential), Obesity, Asthma, Diabetes Mellitus (Noninsulin-Dependent), Schizophrenia, Neural Tube Defects (Folate-Sensitive), Pheochromocytoma (Susceptibility To), Breast Cancer, Glycine Encephalopathy. Two of the databases jointly enriched diseases, including Schizophrenia, Breast Cancer, and Hypertension.

### 3.4 Network pharmacology analysis of PSELNs-BC

The GeneCards database and the OMIM database were used to obtain BC-targets. Among them, 18,182 BC-targets were obtained through the GeneCards database. The data was sorted in descending order based on the relevance score, and the top 2,000 targets were selected. And 513 BC-targets were obtained through the OMIM database. The target genes obtained from the OMIM database and GeneCards database were merged to remove duplicates, and 2,369 BC-targets were obtained.

**FIGURE 4:**
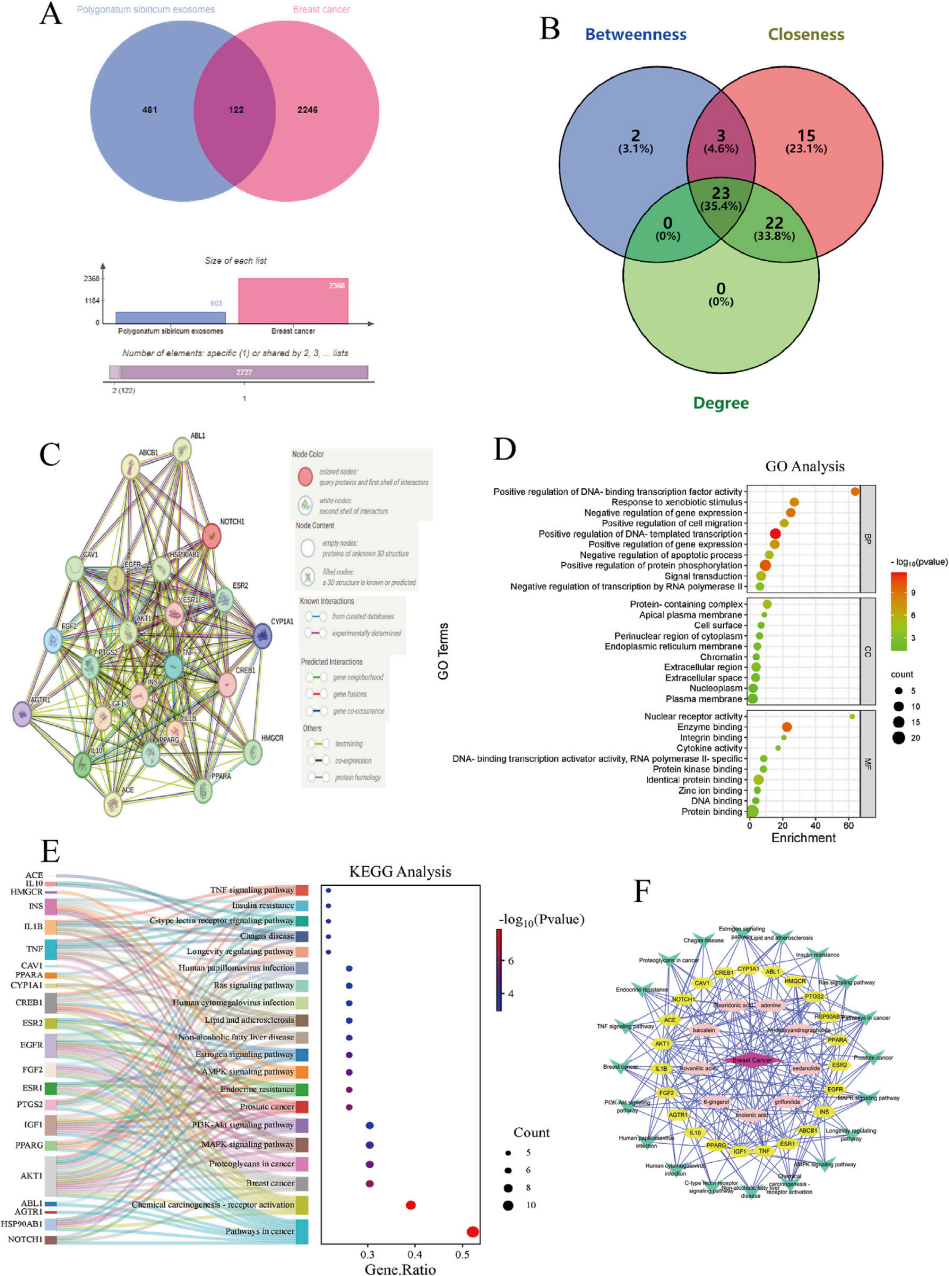
Network pharmacology analysis. **(A,B)** PSELNs-targets and BC-targets; **(C)** PPI network; **(D)** GO analysis; **(E)** KEGG analysis; **(F)** Active metabolite-target-pathway-disease network diagram.

The 23 active metabolites of the PSELNs were enriched for disease targets in the MATMAN-TCM database, and a total of 603 targets were identified from the active metabolites. These targets could be considered as the targets of PSELNs. The BC-targets and PSELNs-targets were combined, and intersection targets were taken to obtain 122 common targets, which were potential targets of PSELNs against BC (Figure 4A). The interaction network of 122 targets was obtained through the STRING database, and Cytoscape software (3.10.1) was used to obtain key targets. As shown in Figure 4B, a total of 23 key targets were identified based on the centrality values of Betweenness (122.6393), Closeness (0.0042), and Degree (20.7377). These key targets mainly include IL1B, PPARG, HMGCR, CAV1, AKT1, TNF, ESR1, etc., 23 key targets were analyzed for their interaction relationships in the STRING database using “*Homo sapiens*” as the research object, and a PPI network was obtained (Figure 4C). There was a total of 23 nodes (network nodes representing proteins) and 196 edges (edges representing protein-protein associations), with an average node degree of 17, and PPI enrichment *p*-value <1.0 e−16.

GO and KEGG analyses were used to explore the potential mechanism of the PSELNs against BC. GO enrichment analysis was performed at BP, MF, and CC levels, and the top 10 results were shown in Figure 4D. Within the BP category, the target proteins were primarily involved in the positive regulation of DNA-templated transcription, the positive regulation of protein phosphorylation, and the negative regulation of gene expression. In the CC class, the target proteins were categorized as protein-containing complex, plasma membrane, nucleoplasm, etc. The MF class target proteins were mainly involved in enzyme binding, protein binding, and identical protein binding. KEGG analysis enriched the key signal pathways of the PSELNs against BC, as shown in Figure 4E, including the MAPK signaling pathway, PI3K-AKT signaling pathway, and AMPK signaling pathway. The active metabolite-target-pathway-disease network diagram is shown in Figure 4F.

### 3.5 Bioinformatics analysis

**FIGURE 5:**
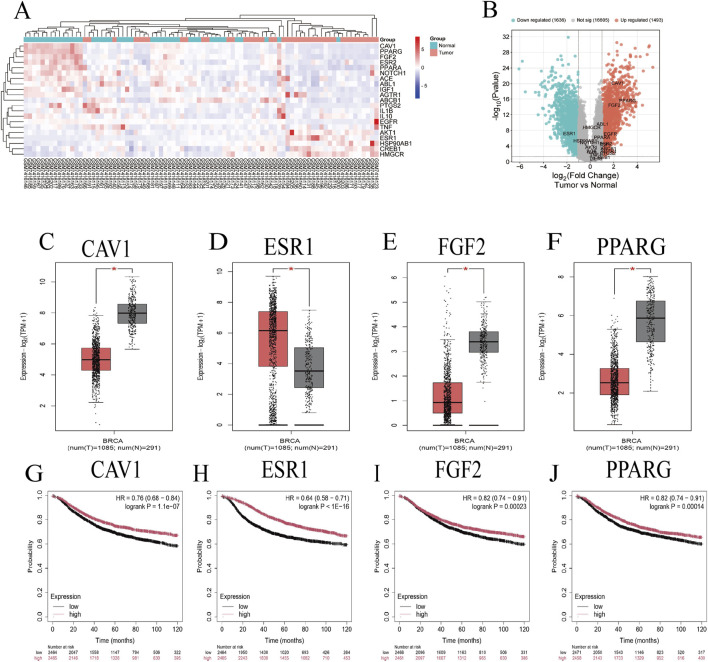
**(A)** Volcanic map of differential genes in BC tissue samples (n = 42); **(B)** Heat map of differentially expressed genes; **(C–F)** analysis of gene expression levels; **(G–J)** survival analysis diagram. (T = Tumor, N = Normal).

In the GEO database, gene chip GSE233242 was the target chip, and the differentially expressed genes of BC tissue samples and paracancerous tissue samples were shown in Figure 5A. The 23 key targets screened by network pharmacology were searched in the gene chip, and 21 genes were found to have differential expression (Figure 5B). The key targets were screened under the conditions of *P* < 0.05 and |log FC| ≥ 1.5, and only 4 genes showed significant differences in expression, namely, Caveolin-1 (CAV1), Estrogen-receptor one (ESR1), Fibroblast growth factor 2 (FGF2) and peroxisome proliferator-activated receptor gamma (PPARG). In the single gene analysis of these 4 genes, as shown in Figures 5C–F, except for ESR1, which was significantly upregulated in BC, CAV1, FGF2, and PPARG were all significantly downregulated in BC. Interestingly, in the prognostic analysis of these 4 genes, their expression levels were positively correlated with survival status (Figures 5G–J).

### 3.6 Molecular docking

**FIGURE 6:**
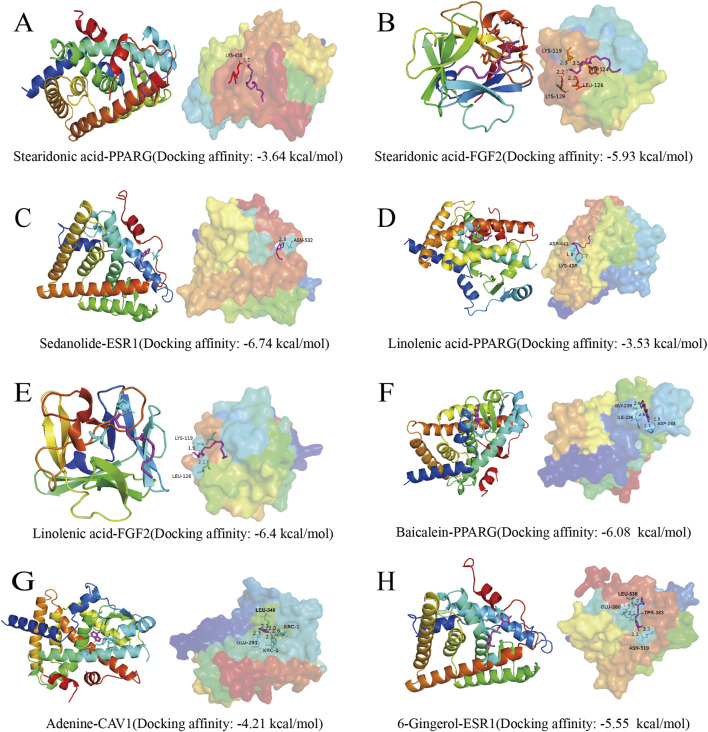
Molecular docking.

Based on the screened core targets, 6 small-molecule compounds were found to bind to the active metabolites. A molecular docking binding energy of less than 0 indicates that small molecules can spontaneously bind to proteins, while a binding energy of less than −5 kcal/mol indicates that the binding between small molecules and proteins is relatively stable (García Molina et al., 2023; Li et al., 2022). The docking results were shown in Figures 6A–H, indicating that 5 active metabolites can spontaneously bind to core targets. Among them, Stearidonic acid can stably bind to FGF2, Sedanolide can bind to ESR1, Linolenic acid can bind to FGF2, Baicalein can bind to PPARG, and 6-Gingerol can stably bind to ESR1.

### 3.7 Molecular dynamic simulation

**FIGURE 7:**
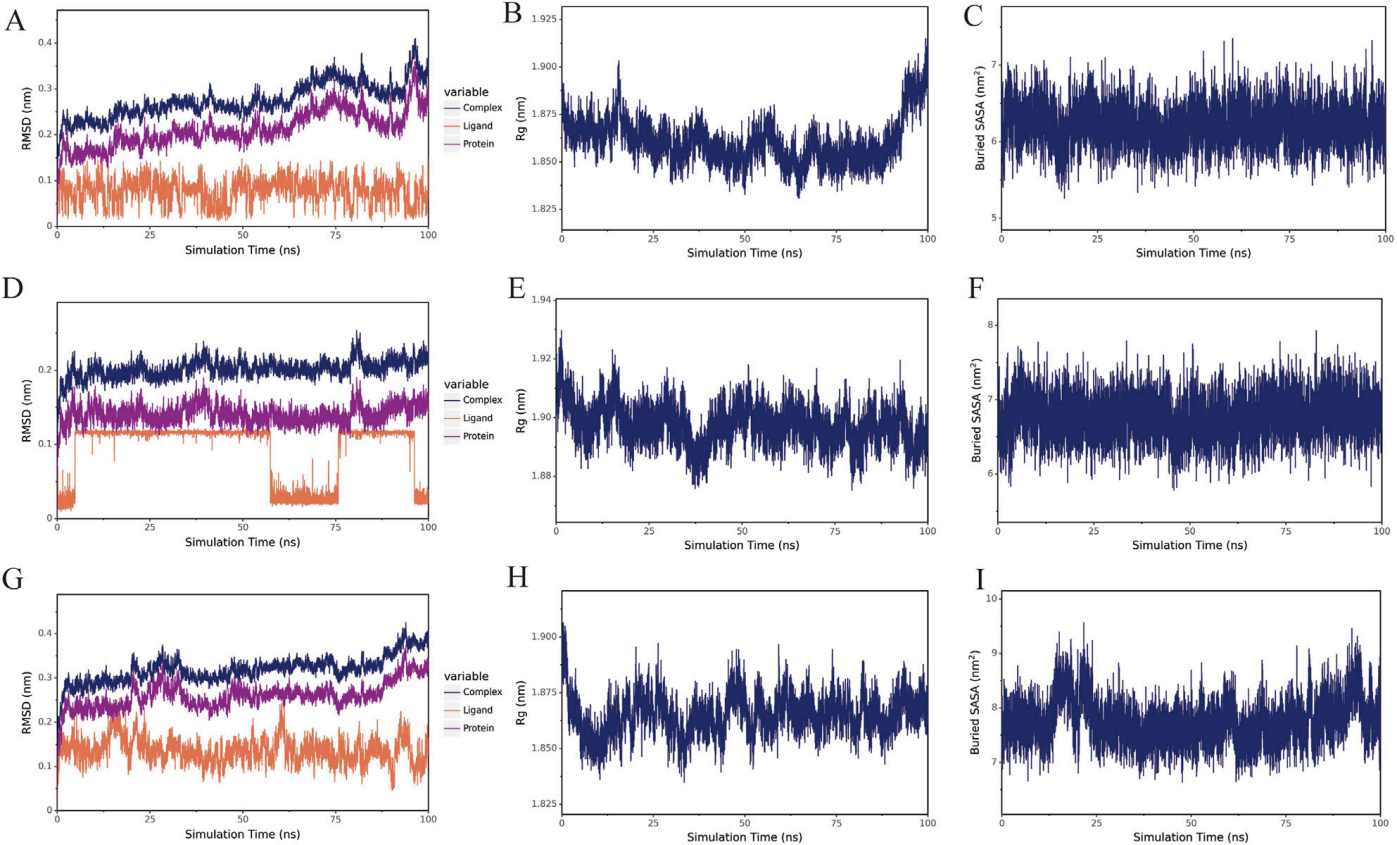
The results of the MD simulation. **(A–C)**: RMSD analysis, Rg analysis, and Buried SASA analysis of Sedanolide-ESR1 in sequence; **(D–F)**: RMSD analysis, Rg analysis, and Buried SASA analysis of Baicalein-PPARG in sequence; **(G–I)**: RMSD analysis, Rg analysis, and Buried SASA analysis of 6-Gingerol-ESR1 in sequence.

MD simulation was carried out on the composite structure with relatively stable binding in the molecular docking results, through Root Mean Square Deviation (RMSD) analysis, Radius of Gyration (Rg) analysis, and Buried Solvent Accessible Surface Area (Buried SASA) analysis. It was found that the binding of sedanolide and ESR1, Baicalein and PPARG, and 6-Gingerol and ESR1 can remain stable, as shown in Figure 7.

### 3.8 *In vitro* validation of CCK-8 method

**FIGURE 8:**
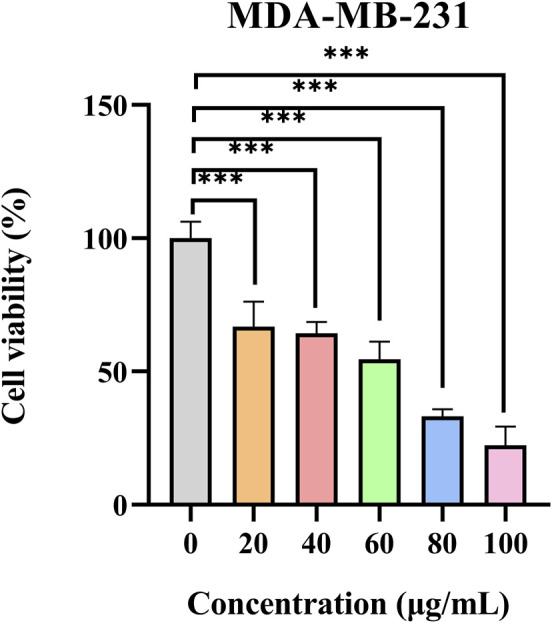
Survival rate of MDA-MB-231 cells at 24 h under different concentrations of the PSELNs (n = 5, ***P < 0.001).

The CCK-8 method was used to study the effect of the PSELNs on the proliferation of breast cancer MDA-MB-231 cells. As shown in Figure 8, the survival rate of MDA-MB-231 cells was significantly reduced after intervention with the PSELNs of different concentrations (*P* < 0.001). This preliminarily confirmed that the PSELNs have significant anti-BC activity *in vitro*, which was consistent with the results of our previous bioinformatics analysis.

## 4 Discussion

In recent years, the research on exosomes has spread from human sources to plant sources, and more and more researchers have begun to pay attention to PELNs. In addition to botanists focusing on the information regulatory role of PELNs in the plant itself, more medical scholars are exploring their enormous potential in the field of medicine, mainly including their direct application in disease treatment and as drug carriers (Dad et al., 2021). The PELNs have their unique advantages, such as easy accessibility, non-immunogenic, exceptional biocompatibility, biodegradability, and capability to bypass biological barriers, etc (Han et al., 2022; Zhu et al., 2023). The most fundamental aspect of studying PELNs is their extraction and separation. Currently, the most commonly used method is the ultracentrifugation method (Ou et al., 2023). In this study, we extracted the PSELNs using ultracentrifugation and characterized the extracted PSELNs using TEM, NTA, and the Zeta potential.

Currently, omics technologies are extensively applied in the study of PELNs. For instance, Chen et al. utilized lipidomics and metabolomics to analyze exosome-like nanoparticles derived from tea leaves, revealing their richness in phosphatidylcholine, phosphatidylmethanol, polyphenols, and flavonoid compounds (Chen et al., 2023). After isolating the PSELNs, metabolomics based on LC-MS/MS was used for preliminary analysis of their components. The results showed that the most abundant components in the PSELNs were various lipid molecules, including prenol lipids (25.51%), fatty acyls (11.9%), steroids and steroid derivatives (8.5%). Lipids were essential components of lipid bilayer structures of the PELNs (Dhar et al., 2024). And lipids are crucial for the stability, uptake, and other biological functions of the PELNs. In addition, components such as leucine, baicalein, linolenic acid, adenine, etc., were detected in the PSELNs. This was the first time that the PSELNs have been extracted and analyzed for their composition, but there were also significant limitations in this work, such as not analyzing nucleic acid molecules such as miRNA through other technical means. Overall, the analysis of the PSELNs components was still not comprehensive enough. However, the detected metabolites laid a certain foundation for the subsequent study of the pharmacological activity of the PSELNs.

Given the complexity of TCM ingredients and their multi-target approach to disease treatment, network pharmacology is frequently employed in research on TCM’s therapeutic applications (Hu et al., 2023; Liu T. et al., 2024). Due to the complexity of the PELN’s components, we have applied network pharmacology for the first time to predict their pharmacological activity. After screening the metabolites detected by the metabolomics, the diseases that the PELNs may treat were predicted based on the active metabolites, mainly including Schizophrenia, Breast Cancer, and Hypertension. There have been studies on BC by *Polygonatum sibiricum*. For example, *P. sibiricum* component liquiritigenin restrains BC cell invasion and migration by inhibiting HSP90 and chaperone-mediated autophagy (Xu et al., 2024). Polysaccharide-rich extract from *P. sibiricum* protects hematopoiesis in bone marrow suppressed by triple-negative BC (Xie et al., 2021). We select BC as the research disease, and continue to study the mechanism of the PSELNs against BC through network pharmacology. The screened active metabolites in the PSELNs, such as linolenic acid and baicalein, have been found to have anti-BC activity. α-linolenic acid can inhibit the migration of human triple-negative BC cells by reducing the expression of Twist1 and inhibiting Twist1-mediated epithelial-mesenchymal transition (Wang et al., 2020). Baicalein induces apoptosis and autophagy of BC cells via inhibiting the PI3K/AKT pathway *in vivo* and *in vitro* (Yan et al., 2018). This further suggests the potential of the PSELNs in anti-BC. KEGG analysis enriched the key signaling pathway of the PSELNs against BC, including the MAPK signaling pathway and PI3K-AKT signaling pathway. The work found that the MAPK signaling pathway and PI3K-AKT signaling pathway play an important role in the occurrence and development of BC, and drugs can play an anti-BC role by regulating them (Liu et al., 2023; Miricescu et al., 2020). For example, Soyasaponin Ag inhibits triple-negative BC progression via targeting the DUSP6/MAPK signaling (Huang et al., 2021). Syringin can play an anti-BC role by regulating PI3K-AKT pathways (Wang et al., 2022). This suggests that the MAPK signaling pathway and PI3K-AKT signaling pathway may play an important role in the anti-BC effect of the PSELNs.

Through network pharmacology analysis, 23 key targets of the PSELNs against BC were screened, including PPARG, ESR1, FGF2, CAV1, etc. Gene chip analysis of key genes revealed that CAV1, FGF2, and PPARG exhibited lower expression in BC tissue samples compared to adjacent non-cancerous samples, whereas ESR1 showed higher expression. Notably, the expression levels of all 4 core proteins were positively associated with survival outcomes. These targets play an important role in the occurrence and development of BC. For example, PPARG may reduce the development of BC by regulating the immune microenvironment (Li et al., 2023; Wu et al., 2021). FGF2 from cancer-associated fibroblasts stimulates the growth and progression of human BC cells through FGFR1 signaling (Suh et al., 2020). Tumor-derived CAV1 promotes pre-metastatic niche formation and lung metastasis in BC (Wang Y. et al., 2023). ESR1 in BC is a very important target, and ESR1 mutation plays an important role in drug response and disease prognosis (Kingston et al., 2024; Dustin et al., 2019). Molecular docking technology was used to further validate the binding of these 4 core targets to active metabolites in the PSELNs. Among them, Stearidonic acid binds well to FGF2, Sedanolide binds well to ESR1, Linolenic acid binds well to FGF2, Baicalein binds well to PPARG, and 6-Gingerol binds well to ESR1. In further MD simulations, Sedanolide and ESR1, Baicalein and PPARG, 6-Gingerol and ESR1 can maintain stable binding. The final cell experiment confirmed the inhibitory effect of the PSELNs on BC. However, although this work has preliminarily validated the composition and anti-tumor pharmacological activity of the PSELNs, further *in vivo* and *in vitro* experiments are still needed for exploration.

## 5 Conclusion

This study used ultracentrifugation to extract the PSELNs and characterized them using TEM, NTA, and the Zeta potential. Then, through proteomics and metabolomics analysis of the PSELNs, a total of 18 proteins and 357 metabolites were identified. The targets of active metabolites were used to predict the anti-BC activity of the PSELNs. Network pharmacology analysis shows that the anti- BC mechanism of the PSELNs involves MAPK signaling pathway and PI3K-AKT signaling pathway. Molecular docking and MD simulations have confirmed that the anti-BC effect of PSELNs may be related to the binding of Sedanolide and ESR1, Baicalein and PPARG, 6-Gingerol and ESR1. The inhibitory effect of the PSELNs on BC was verified by the CCK-8 method. This study analyzed the components of PSELNs and preliminarily explored their potential pharmacological activities in anti- BC, providing a new direction for the development and utilization of *P. sibiricum* resources from the perspective of exosome-like nanoparticles.

## Data Availability

The raw data supporting the conclusions of this article will be made available by the authors, without undue reservation.
