# Supplementary material for: Understanding mechanisms of Polygonatum sibiricum-derived exosome-like nanoparticles against breast cancer through an integrated metabolomics and network pharmacology analysis
Source: Front Chem. 2025 Jun 6;13:1559758. doi: 10.3389/fchem.2025.1559758 (PMC12179076; doi:10.3389/fchem.2025.1559758)
Supplement: Supplementary file 1 [file DataSheet1.zip › raw data/Fig.2/NTA.pdf]

NANOSIGHT

3 2024-06-05 11-48-13

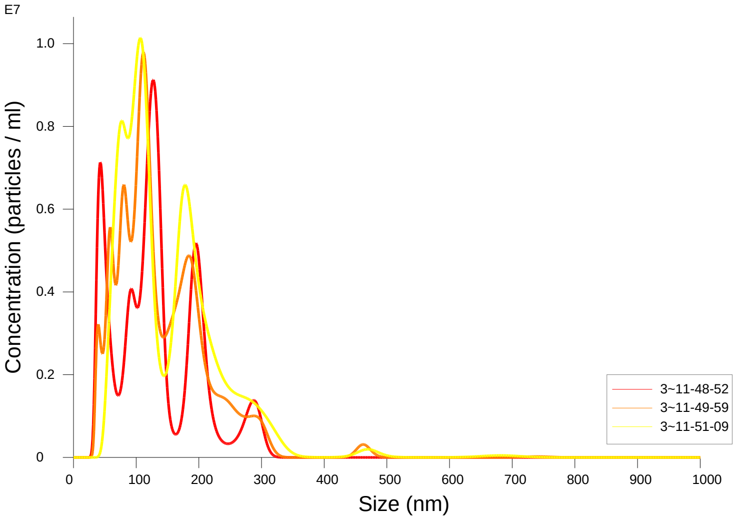

FTLA Concentration / Size graph for Experiment:
3 2024-06-05 11-48-13

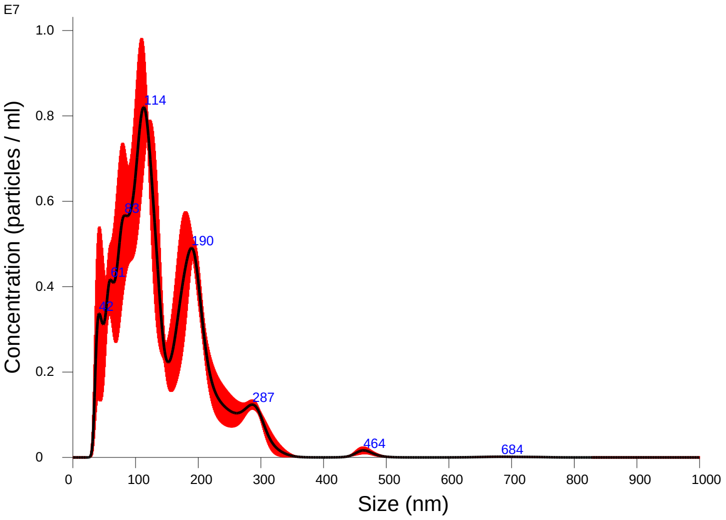

Averaged FTLA Concentration / Size for Experiment:
3 2024-06-05 11-48-13
Error bars indicate + / -1 standard error of the mean

Included Files

3 2024-06-05 11-48-52
3 2024-06-05 11-49-59
3 2024-06-05 11-51-09

Details

NTA Version:	NTA 3.4 Build 3.4.003
Script Used:	SOP Standard Measurement 11-48-13AM 05J~
Time Captured:	11:48:13 05/06/2024
Operator:	
Pre-treatment:	
Sample Name:	3
Diluent:	
Remarks:	

Capture Settings

Camera Type:	sCMOS
Laser Type:	Red
Camera Level:	7
Slider Shutter:	165
Slider Gain:	15
FPS	25.0
Number of Frames:	1498
Temperature:	7.5 ^{o}C
Viscosity:	(Water) 1.4 cP
Dilution factor:	Dilution not recorded

Analysis Settings

Detect Threshold:	5
Blur Size:	Auto
Max Jump Distance:	Auto: 11.1 - 13.0 pix

Results

Stats: Merged Data

Mean:	142.4 nm
Mode:	113.0 nm
SD:	74.3 nm
D10:	62.2 nm
D50:	123.7 nm
D90:	236.7 nm

Stats: Mean +/- Standard Error

Mean:	141.4 +/- 5.4 nm
Mode:	115.1 +/- 6.2 nm
SD:	72.9 +/- 3.8 nm
D10:	60.1 +/- 7.5 nm
D50:	123.7 +/- 1.7 nm
D90:	232.8 +/- 10.9 nm
Concentration:	9.55e+08 +/- 9.81e+07 particles/ml
	52.2 +/- 5.4 particles/frame
	99.8 +/- 2.4 centres/frame

3 2024-06-05 11-48-13

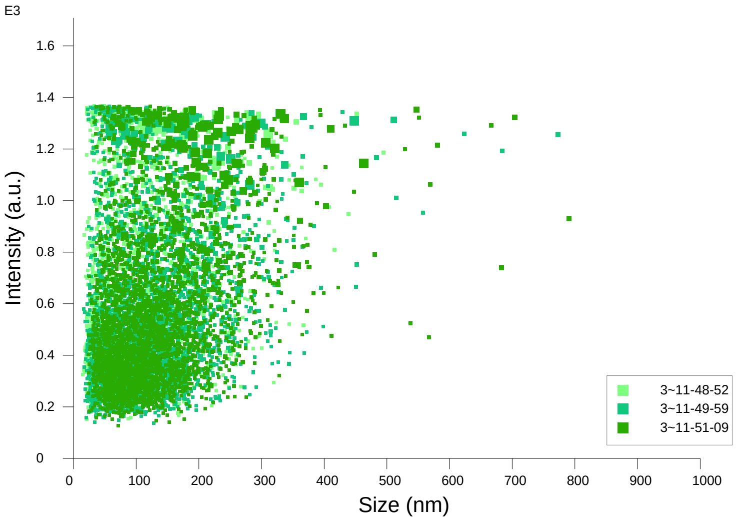

Intensity / Size graph for Experiment:
3 2024-06-05 11-48-13

Script Used: (Full Text):

SOP Standard Measurement 11-48-13AM 05Jun2024.txt
