# Supplementary figures and images for: Understanding mechanisms of Polygonatum sibiricum-derived exosome-like nanoparticles against breast cancer through an integrated metabolomics and network pharmacology analysis
Source: Front Chem. 2025 Jun 6;13:1559758. doi: 10.3389/fchem.2025.1559758 (PMC12179076; doi:10.3389/fchem.2025.1559758)

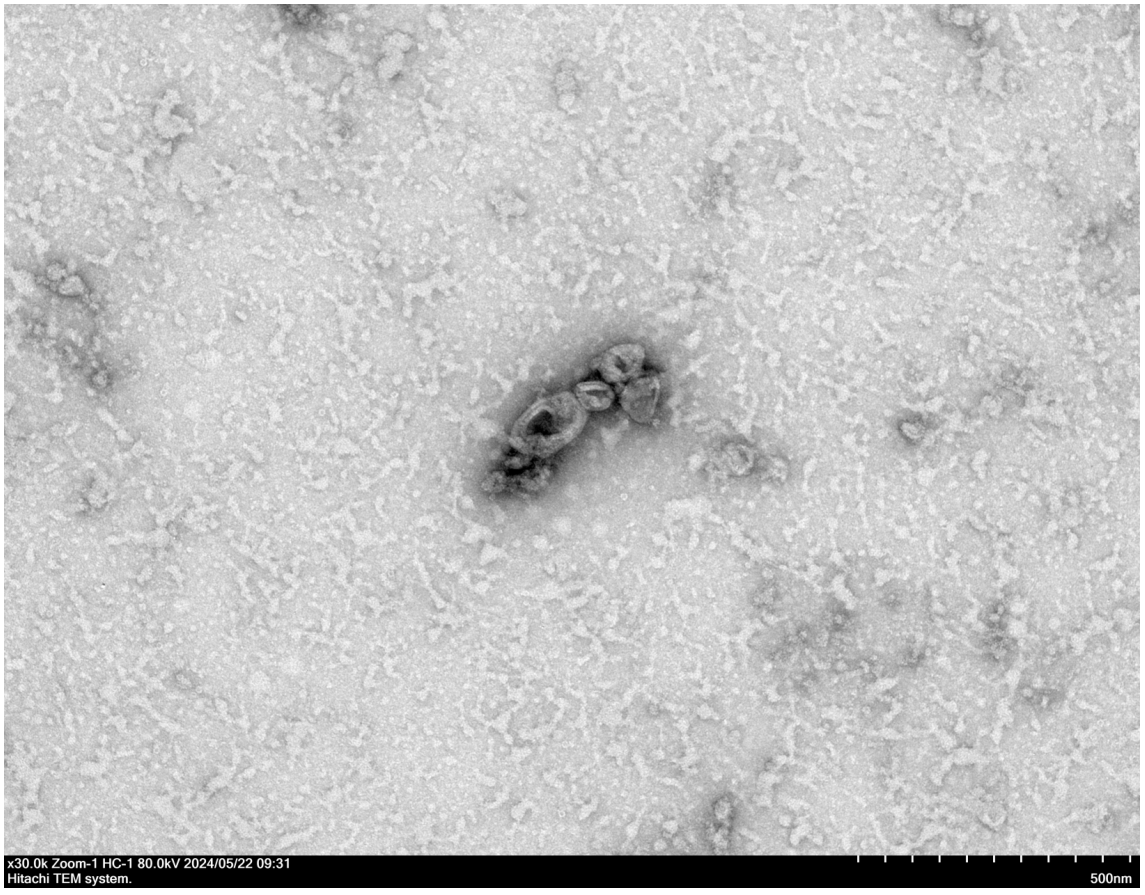

Supplement: Supplementary file 1 [file DataSheet1.zip › raw data/Fig.2/TEM.png]

# Disease Analysis

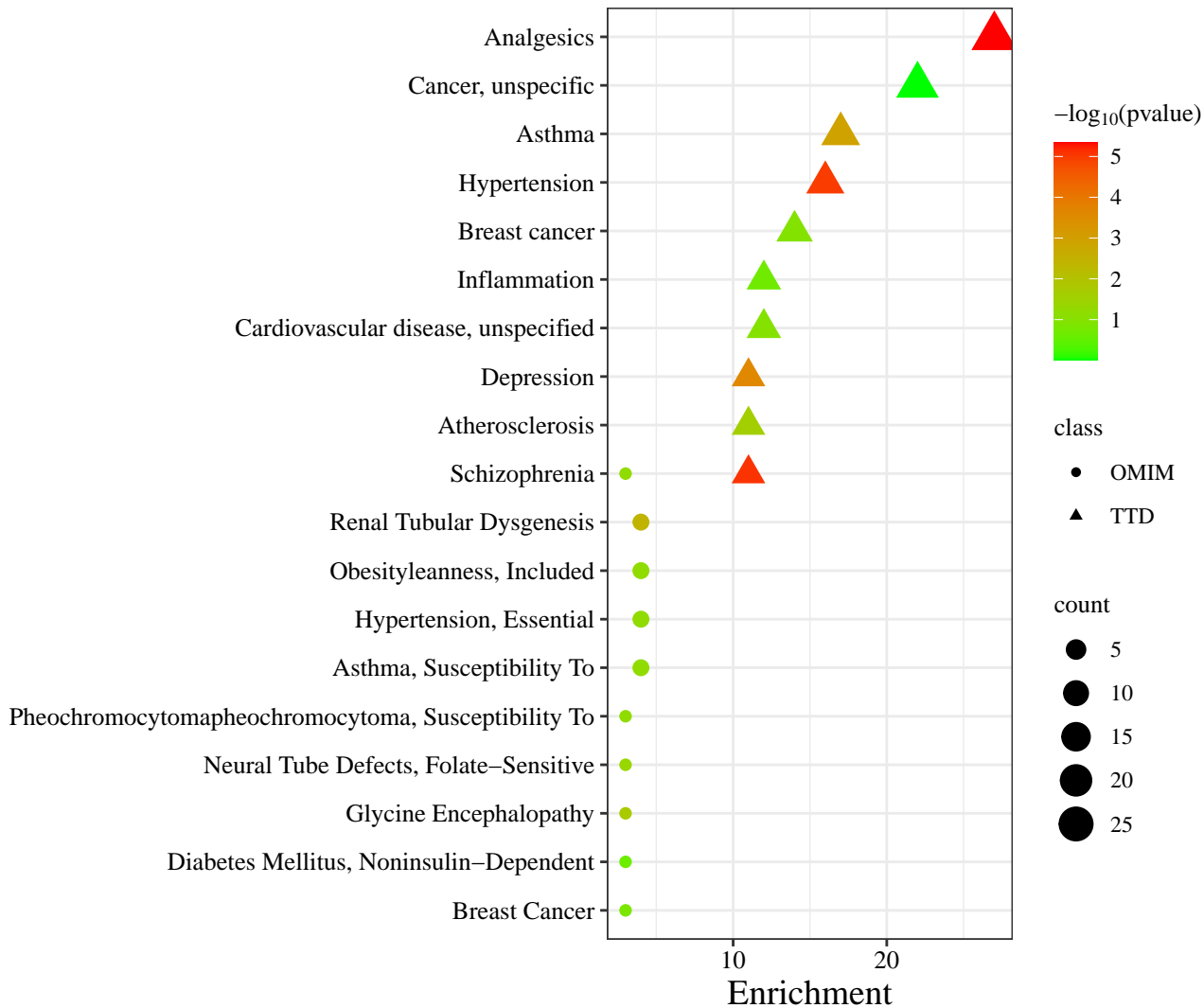

Supplement: Supplementary file 1 [file DataSheet1.zip › raw data/Fig.3/Disease enrichment/1fb20005e45ec7c7.pdf]

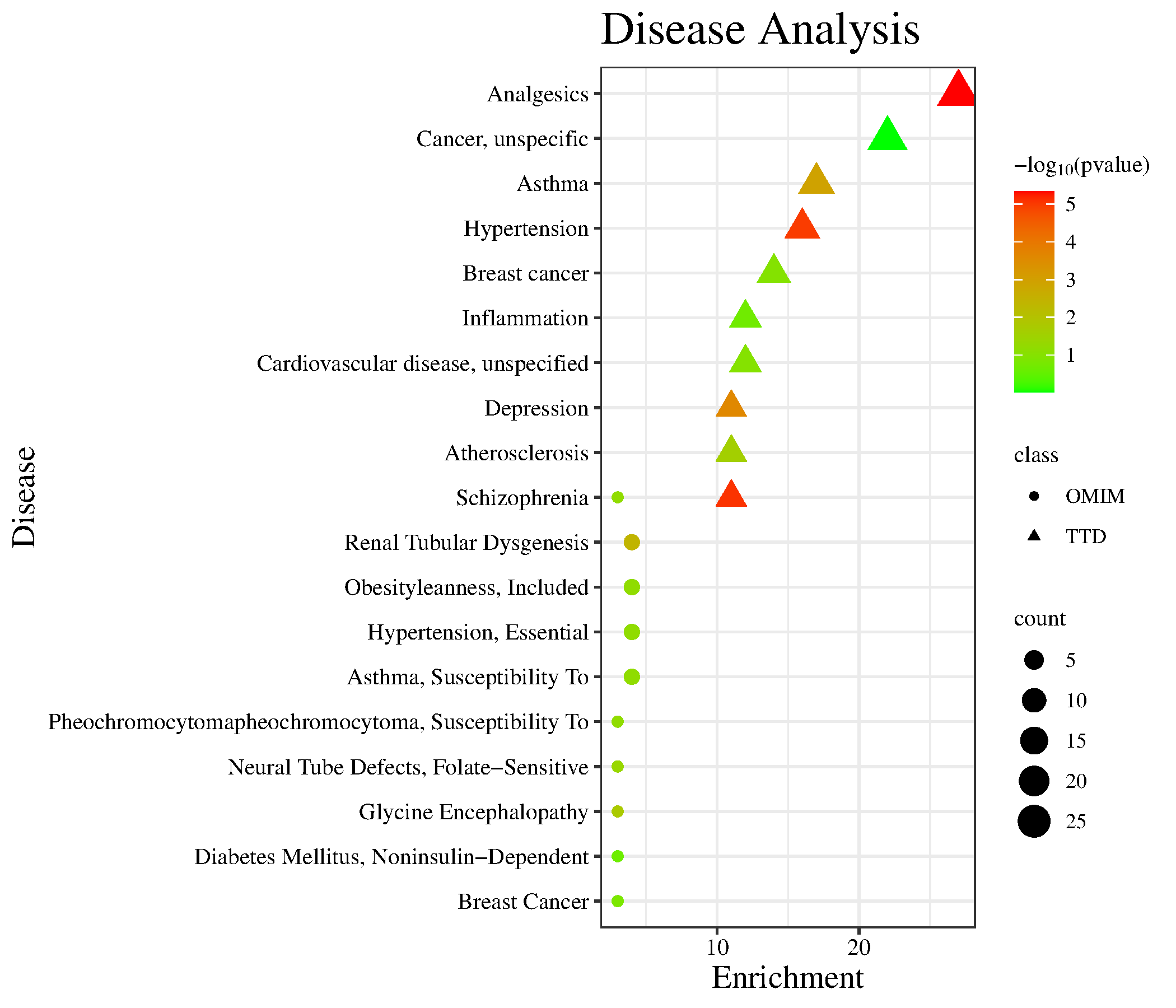

Supplement: Supplementary file 1 [file DataSheet1.zip › raw data/Fig.3/Disease enrichment/1fb20005e45ec7c7.png]
